# Resting heArt and respIratory rates in dogs in their natural environment: new insights from a long-term, international, prospective study in a COhort of 703 dogs using a biometric device for LongitudinaL non-invasive cARdiorespiratory monitoring (the AI-COLLAR study)

**DOI:** 10.3389/fvets.2025.1667355

**Published:** 2025-09-15

**Authors:** Valérie Chetboul, Eric Humbert, Louis Dougoud, Guillaume Lorre

**Affiliations:** ^1^École Nationale Vétérinaire d’Alfort, Maisons-Alfort, France; ^2^INSERM, IMRB, Univ Paris Est Créteil, Créteil, France; ^3^Invoxia, Issy-les-Moulineaux, France

**Keywords:** artificial intelligence, cardiology, canine, heart disease, cardiac physiology

## Abstract

**Background:**

Wearable devices are increasingly used in human medicine to monitor various cardiovascular parameters and support heart health. Similar tools have recently emerged in veterinary medicine. However, their current limitation is the lack of large-scale data in healthy animals, a prerequisite for identifying potentially pathological variations using artificial intelligence (AI)-based algorithms.

**Objectives:**

To establish a large database of resting heart rate (HR) and respiratory rate (RR) recorded over extended periods using a commercially available biometric health-monitoring device in a large international cohort of apparently healthy (AH) dogs.

**Animals:**

703 AH dogs (median age [interquartile range] = 3.8 years [2.2–7.2]; body weight = 23.0 kg [14.8–31.8]).

**Methods:**

Prospective observational study (2022–2025) including AH dogs of any age, breed, and sex, provided owners confirmed their dog’s apparent good health via a dedicated questionnaire.

**Results:**

Median device wear time was 189.0 days [51.0–433.0]. Both HR and RR significantly decreased early in life, then stabilized, with a slight increase in older dogs. Both were also lower at night than during the day (*p* < 0.0001). In dogs living in the Northern Hemisphere, HR and RR showed opposite significant seasonal patterns. Effects of sex, weight and breed were also analyzed.

**Conclusion and clinical importance:**

This unique large-scale biometric study provides real-world reference data on resting HR and RR in dogs and the influence of intrinsic (age, sex, weight, breed) and extrinsic (circadian rhythm, season) factors on these vital signs, thus offering new insights into canine cardiorespiratory physiology under natural conditions, and laying the foundation for future AI-based detection of abnormal patterns.

## 1 Introduction

In human medicine, including the cardiovascular field, digital technologies are playing an increasingly prominent role in healthcare, a paradigm shift that was markedly accelerated by the COVID-19 pandemic (1–4). One notable consequence of the crisis was the widespread and rapid adoption of remote consultations, underscoring the growing demand for digital health solutions. This digital evolution has led to the emergence of new terminology, including the concept of eHealth, also referred to as digital or connected health, which is defined by the World Health Organization as the use of information and communication technologies for health and health-related fields (5). eHealth encompasses a broad range of domains, among which mHealth refers specifically to the practice of medicine supported by mobile devices such as smartphones and tablets, often in combination with dedicated health applications and wearable technologies (5). mHealth is undergoing exponential growth, largely driven by the increasing global use of smartphones in everyday life: the number of smartphone users worldwide is currently estimated at 4.9 billion, representing approximately 60% of the global population (6).

Among the many innovations in digital health, wearable devices, including smartwatches, biometric patches, and chest or wrist monitors, have gained significant traction not only in the domain of wellness, but also in cardiovascular medicine. These biometric tools enable non-invasive, real-time monitoring of “surface” physiological parameters such as heart rate (HR), heart rhythm (via electrocardiogram tracings), respiratory rate (RR), and also physical activity (3, 4, 7–11). Their growing adoption is further supported by marked advances in artificial intelligence (AI), offering the interpretation of large volumes of longitudinal physiological data. This synergy paves the way for more personalized, preventive, and predictive cardiovascular care, as emphasized in recent consensus statements by the European Society of Cardiology (ESC) Working Group on e-Cardiology and other affiliated cardiology associations (3, 4).

This rapidly evolving digital healthcare industry has also expanded into the veterinary field, where similar wearable devices for animals, such as smart dog collars, have recently become commercially available (12–16). However, their current limitation lies in the lack of large-scale reference data from healthy animals, which is essential for the reliable detection of potentially pathological deviations using AI-based algorithms.

The main objective of this prospective observational study was therefore to establish, for the first time, a long-term, non-invasive reference database of resting HR and RR values, derived from intermittent high-frequency recordings using a commercially available biometric health-monitoring device (i.e., Invoxia Biotracker GPS for Dogs®, Invoxia, Issy-les-Moulineaux, France) in a large international cohort of apparently healthy (AH) dogs monitored in their natural home environment.

## 2 Materials and methods

This prospective observational study was conducted between 2022 and 2025 using data extracted from the Invoxia database, based on dogs equipped with Invoxia Biotracker GPS for Dogs®. Dogs were enrolled according to pre-specified inclusion and exclusion criteria and followed over time, with data collected longitudinally under real-life conditions, without any experimental intervention. At activation of the device, owners accepted Invoxia’s terms of service and privacy policy, which explicitly allow the use of fully anonymized, aggregated data for analytical and research purposes. Invoxia retains legal ownership of these anonymized data. The analysis performed in the present study was therefore based on fully anonymized, passively collected data and did not require additional ethical approval or specific owner consent.

### 2.1 Cardiorespiratory monitoring device

Resting HR and RR data were collected using Invoxia Biotracker GPS for Dogs® (initially launched as Minitailz), a non-invasive, collar-mounted tracker developed for long-term physiological monitoring in dogs, which received the Consumer Electronics Show (CES) Innovation Award for Best AI Innovation in 2024 (Las Vegas, NV, USA).

This wireless biometric health tracker enables repeated, non-invasive, and autonomous monitoring of resting HR and RR in dogs throughout the day and night. Measurements are automatically recorded only when the dog is lying down and immobile - whether awake or asleep - for at least 40 s, ensuring that the data reflect true resting physiology. They rely on a technique known as seismocardiography, inspired by the analysis of seismic activity. This method records subtle mechanical vibrations generated by cardiac contractions and respiratory movements at the level of the neck. The signals are then transmitted and analyzed using AI-based algorithms, which identify heartbeats and respiratory cycles to compute HR and RR values, even in dogs with thick coats, and without the need for shaving, electrodes, or physical restraint (16).

The device performs regular recordings lasting between 1 and 3 min, spaced at minimum 15-min intervals. These intermittent high-frequency measurements provide up to 120 min of usable data per day, over 10–15 consecutive days, thanks to the device’s multi-day battery life.

The accuracy of the measurements has been validated at 99.6% for HR and 98.6% for RR, regardless of the dog’s breed, age, body weight, or coat characteristics, compared to portable ECG (for HR) and manual counting from thoracic video recordings (for RR) (16). In addition, individual heartbeats were detected with a temporal resolution < 50 ms, achieving an F1 score of 98.0% (16).

### 2.2 Cohort constitution

Inclusion criteria required that dogs be classified as AH, regardless of country, age, breed, or sex, provided they had worn the biometric health-monitoring device for at least 5 h one day. In addition, four metadata fields (i.e., age, sex, breed, and body weight) had to be reported, and the owner had to confirm the dog’s apparent good health by completing a dedicated health questionnaire. This questionnaire consisted of a checklist of predefined clinical signs (e.g., cardiac, neurological, locomotor), with boxes to tick if any signs were present, as well as an open field for reporting any additional observed clinical abnormalities. Only dogs with no owner-reported clinical signs were classified as AH and included in the study. Dogs with missing or incomplete questionnaires were not included.

Furthermore, among these AH dogs, those that experienced either clinical or statistical events during the monitoring period were excluded from the final study population. Clinical events were defined as any health issues reported by owners and flagged on the digital platform (e.g., death, respiratory distress). Statistical events referred to unusual variations in resting HR or RR, automatically detected by the algorithm. For both HR and RR, we first calculated the daily difference between the current 40-day moving average and the value 40 days earlier. A day was flagged as a statistical event when this difference exceeded ± 3 standard deviations of the cohort-wide difference distribution. Days with < 15 min of valid data were excluded from all moving-average and standard deviation calculations. Any statistical event corresponding to an owner-reported disorder was subsequently reclassified as clinical.

### 2.3 Statistical analysis

Categorical data are reported as proportions or percentages, while continuous data are presented as medians with interquartile ranges [IQR]. All statistical analyses were conducted using Python 3.10.8 (Python Software Foundation, Wilmington DE, USA) with the libraries statsmodels 0.14.4, SciPy 1.15.2, and NumPy 1.26.4.

For each model the null hypothesis of zero slope was tested with two-sided Student t-tests, and normality of the residuals was assessed using Shapiro–Wilk tests and QQ-plots. This supported the use of the parametric OLS framework and Pearson correlation coefficients (r) were reported.

To assess seasonal variation in HR and RR we used linear mixed-effects models with month of the year as a fixed categorical effect (treated as an ordered factor with January as reference) and dog identity as a random intercept, thereby estimating month-specific deviations while accounting for repeated measures.

Comparisons of HR or RR between adult subgroups (male *versus* female, day *versus* night, healthy *versus* diseased dogs) employed Welch’s t-test, after checking the raw distributions with Shapiro–Wilk tests and QQ-plots, which were deemed sufficiently symmetric. All *p* values are two-sided, and values < 0.05 were considered statistically significant.

## 3 Results

### 3.1 Characteristics of the whole study population (*n* = 703 AH dogs)

**Figure 1:**
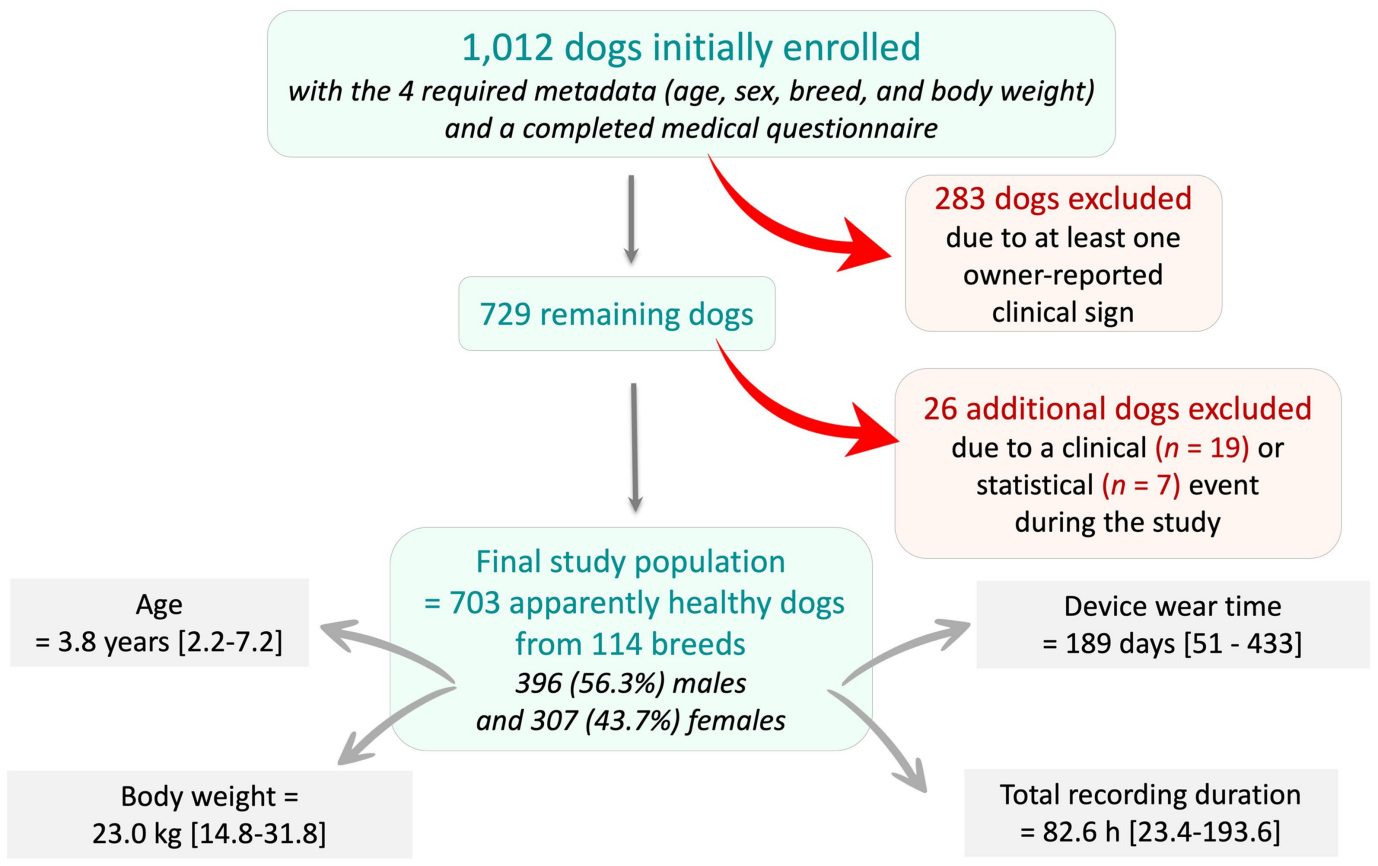
Flowchart illustrating the recruitment process and inclusion criteria for the study population, from an initial cohort of 1,012 dogs with the four required metadata (age, sex, breed, and body weight) and a completed medical questionnaire. Dogs were excluded if they presented at least one owner-reported clinical sign at enrollment (*n* = 283), or if a clinical or statistical (*n* = 26) abnormality occurred during the study period. A total of 703 apparently healthy dogs were therefore included in the final study population.

**Figure 2:**
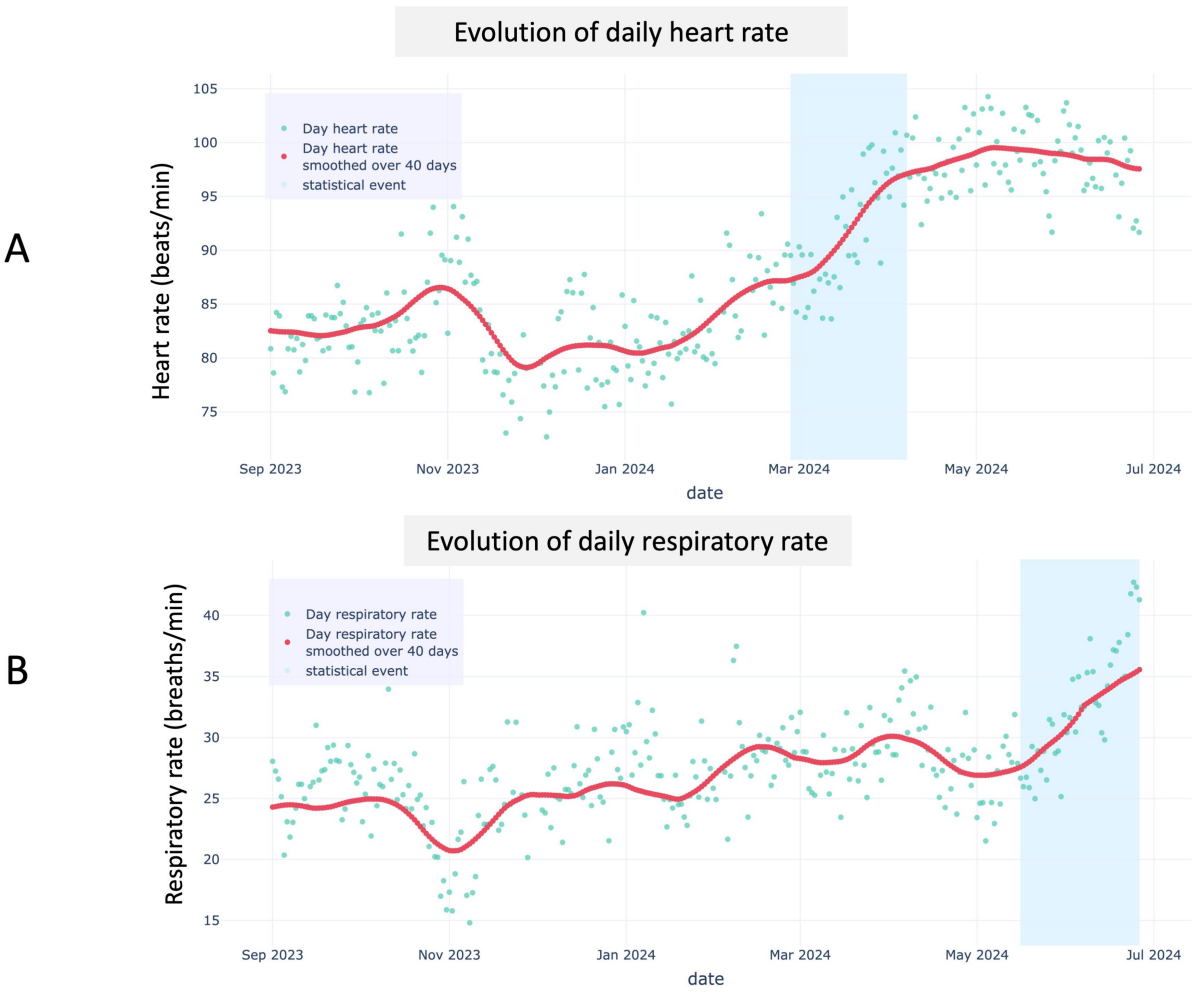
Representative example of algorithmic detection of a statistical event in a dog with degenerative mitral valve disease, monitored at home for heart rate **(A)** and respiratory rate **(B)** using the biotracker. A steady increase in the 40-day moving average of daytime heart rate (red line) was detected by the algorithm **(A)**, with its 40-day difference exceeding the alert threshold approximately 60 days before a similar change was observed in respiratory rate due to congestive heart failure (i.e., pulmonary edema; **B**). In both panels, turquoise dots represent daily means, the red line is the 40-day moving average, and the blue shaded band denotes the period where the statistical events occurred.

A total of 1,012 dogs for which the four required metadata (age, sex, breed, and body weight) were available and the medical questionnaire fully completed were initially enrolled (Figure 1). Among these, 283 dogs were excluded due to at least one owner-reported clinical sign. Of the remaining 729 dogs, 26 were further excluded because of a clinical (*n* = 19) or statistical (*n* = 7) event identified during the study period. Among these 26 excluded dogs, one dog experienced a statistical event that was subsequently reclassified as a clinical event due to owner-reported pulmonary edema related to decompensated degenerative mitral valve disease (DMVD; Figure 2). This congestive heart failure (CHF) event was characterized by an increased RR, which was preceded by a 120-day gradual increase in HR that remained undetected by the owner.

The final population therefore included 703 AH dogs, 396 (56.3%) males and 307 (43.7%) females, with a median age of 3.8 years [IQR: 2.2–7.2] ranging from 0.2 to 18.1 years, and a median body weight of 23.0 kg [14.8–31.8] ranging from 0.9 to 75.0 kg (Figure 1). The biometric health tracker was worn for a median duration of 189 days [51–433], with a median total recording time of 82.6 h [23.4–193.6].

Among the 703 AH dogs included in the final analysis, 212 (30.2%) were mixed-breed and 491 (69.8%) were purebred. Among the 113 pure breeds represented, the 10 most common were as follows: Golden Retriever (*n* = 41, 5.8%), German Shepherd (*n* = 34, 4.8%), Australian Shepherd (*n* = 30, 4.3%), Labrador Retriever (*n* = 30, 4.3%), Siberian Husky (*n* = 24, 3.4%), Border Collie (*n* = 22, 3.1%), Doberman (*n* = 19, 2.7%), Beagle (*n* = 14, 2.0%), Bernese Mountain Dog (*n* = 10, 1.4%), and Belgian Malinois (*n* = 9, 1.3%).

**Figure 3:**
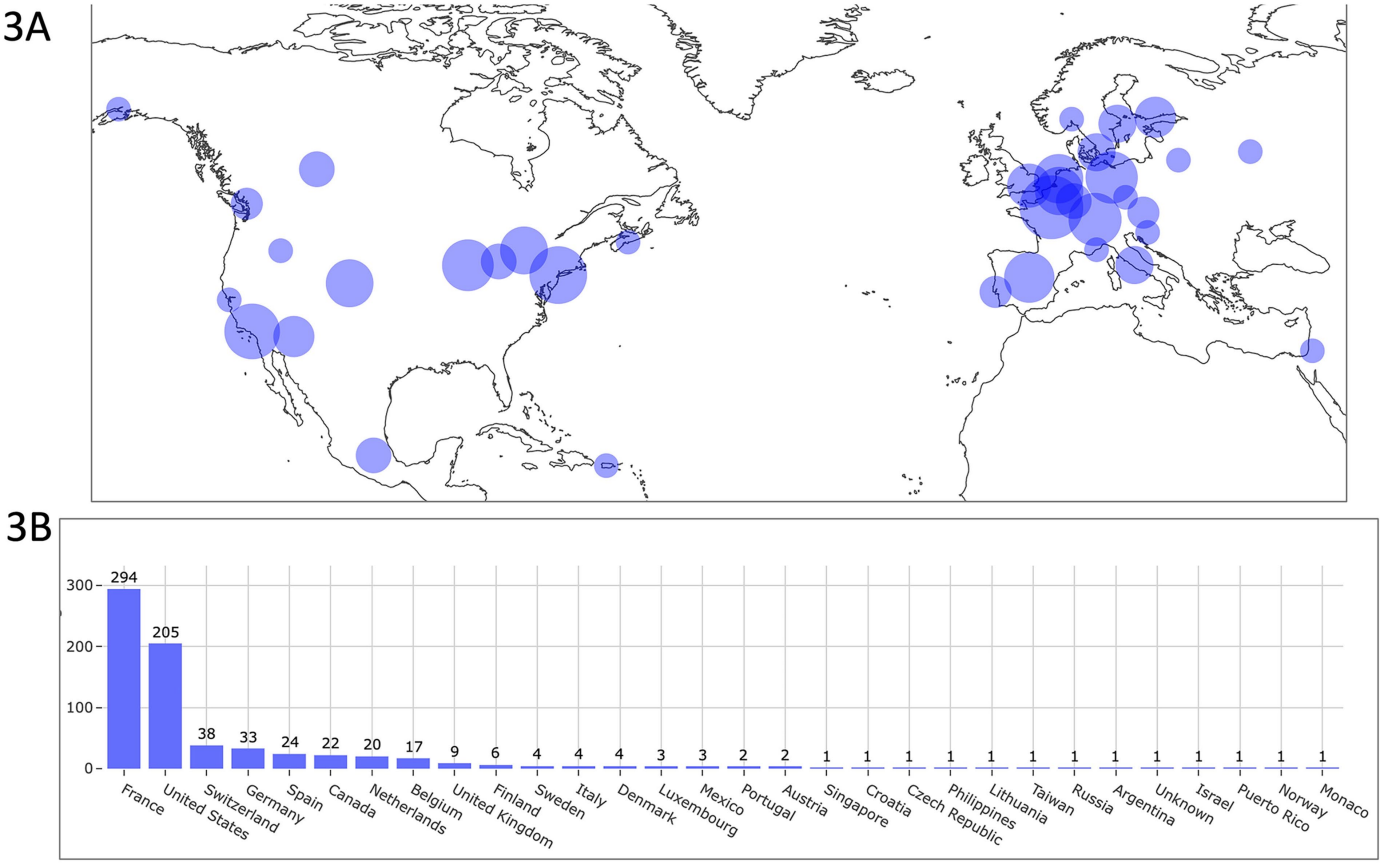
Geographic distribution of dogs equipped with the biometric smart device, shown on a world map **(A)** and as a bar chart **(B)**. Most dogs were located in Europe, with France being the top recruiting country and the United States the second. In **(A)**, circle size is proportional to the number of locally monitored dogs. Some dogs are not represented on the map due to scale limitations (e.g., those located in Taiwan, Singapore, and the Philippines) but are included in the bar chart **(B)**. The map used in Figure 3A is based on data from the Natural Earth dataset (www.naturalearthdata.com), which is in the public domain and free of copyright restrictions.

The 703 AH dogs included in the study were spread across 29 countries (Figure 3), with most located in Europe, and France alone accounting for 41.8% (294/703) of the cases, followed by the United States with 29.2% (205/703).

### 3.2 Effect of age on resting HR and RR in the whole study population (*n* = 703 AH dogs)

**Figure 4:**
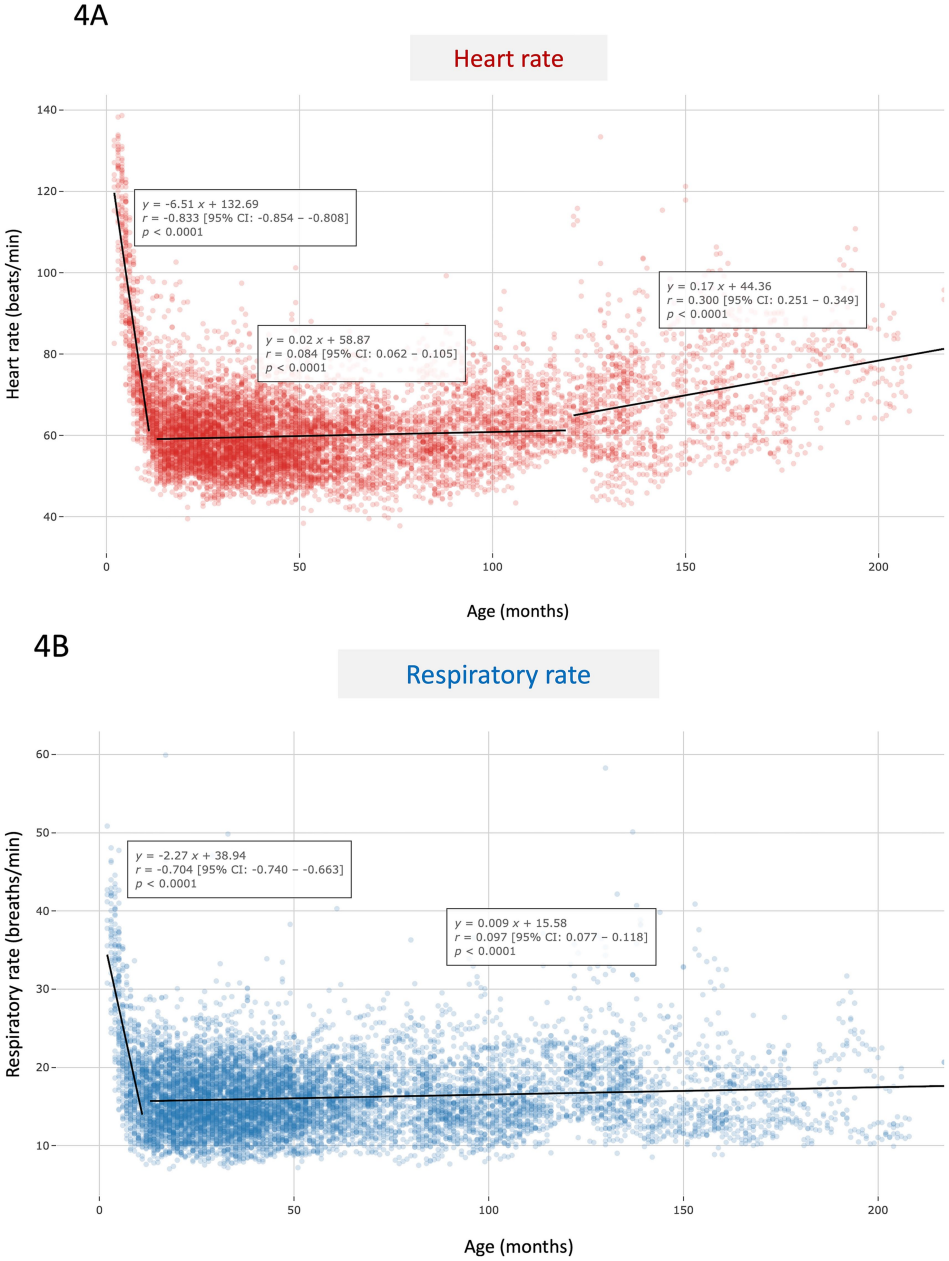
Scatter plot of heart rate **(A)** and respiratory rate values **(B)** according to age (in months) in the whole study population composed of 703 apparently healthy dogs of various breeds. Each dot represents the average heart rate **(A)** or respiratory rate **(B)** per dog and per month of age. Linear regression analyses were performed across age segments, and corresponding equations, correlation coefficients (*r*), 95% confidence intervals, as well as *p-*values are provided.

Average resting HR and RR values per dog and per month of age are presented in Figures 4A,B, respectively. Regarding HR (Figure 4A), a marked and highly significant decrease with age was observed during the first 12 months (*p* < 0.0001; *r* = − 0.833), followed by a relative stabilization during adulthood characterized by a slight increase up to 120 months (*p* < 0.0001; *r* = 0.084), and then another moderate increase between 120 and 220 months (*p* < 0.0001; *r* = 0.300). Thus median HR fell from 78.5 beats per minute (bpm) [69.1–96.8] in puppies (≤ 12 months; *n* = 684 values) to 59.1 bpm [54.1–64.6] in adult dogs (> 1–< 10 years; *n* = 8,145 values) before rising to 68.7 bpm [60.8–77.2] in seniors (≥10 years; *n* = 1,329 values).

Regarding RR (Figure 4B), a marked and highly significant decrease was also observed up to 12 months of age (*p* < 0.0001; *r* = − 0.704), followed by a relative stabilization during adulthood characterized by a slight increase up to 220 months (*p* < 0.001; *r* = 0.097). The corresponding median RR values were 20.1 breaths/min [16.0–25.7] in puppies (≤ 12 months; *n* = 659 values) and 15.7 breaths/min [13.1–18.7] for older dogs (*n* = 9,274 values).

Given these results, and to minimize the confounding effect of age, all subsequent analyses were restricted to the adult non-senior canine group (i.e., *n* = 562 AH dogs aged > 1 year and < 10 years).

### 3.3 Analysis of circadian rhythm, seasons, body weight, and breeds on resting HR and RR in the 562 AH adult dogs (aged > 1 year and < 10 years)

**Figure 5:**
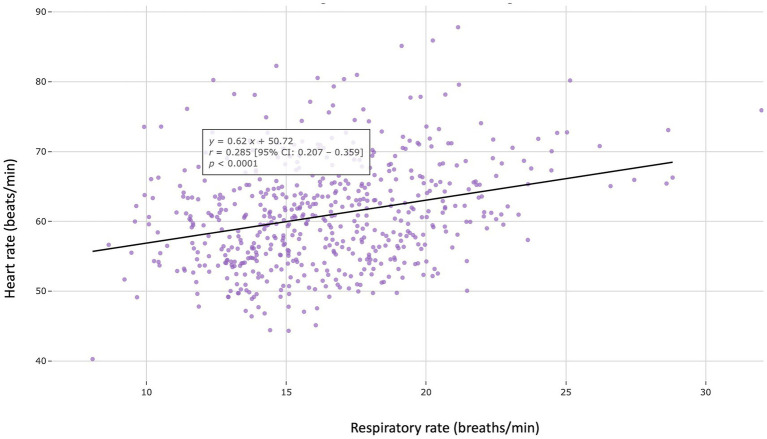
Scatter plot showing a weak but positive correlation between resting heart rate and respiratory rate assessed in the 562 apparently healthy adult dogs (aged > 1 year and < 10 years) included in the study (*r* = 0.285, *p* < 0.0001).

**Figure 6:**
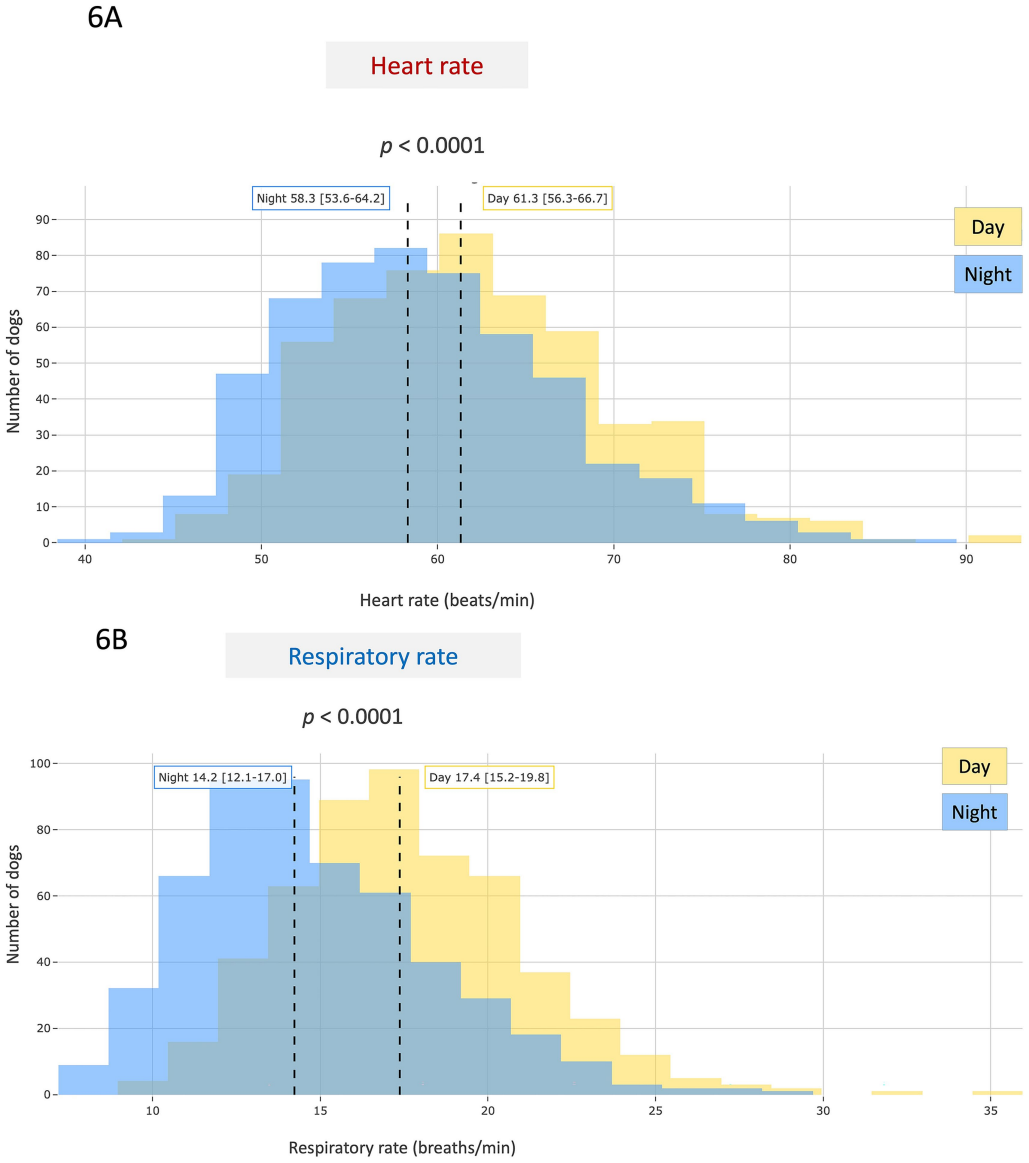
Population distribution of average monthly resting heart rate **(A)** and respiratory rate **(B)** values recorded during daytime (yellow) and nighttime (blue) periods in 533 apparently healthy adult dogs (aged > 1 and < 10 years) out of the 562 included in the study, for whom both day and night measurements were available. Nighttime was defined as the period between midnight and 6:00 a.m., and daytime as the remaining time. Both biometric variables were significantly lower at night (*p* < 0.0001).

**Figure 7:**
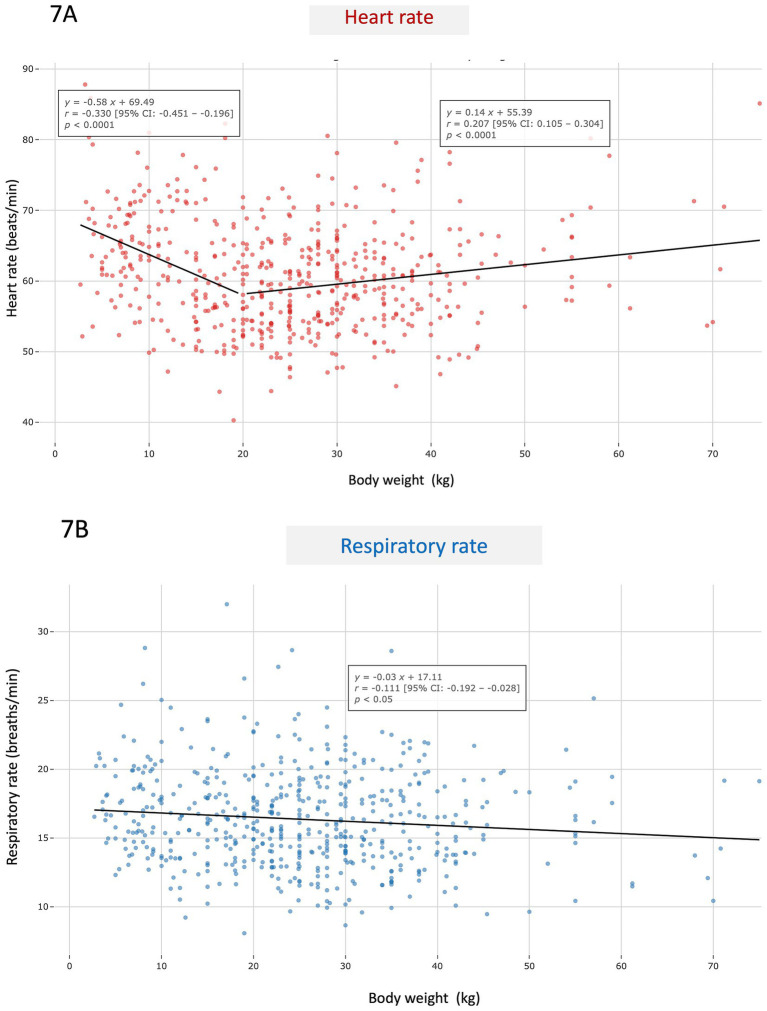
Scatter plot showing average monthly resting heart rate **(A)** and respiratory rate **(B)** values according to body weight (in kg) in the 562 apparently healthy adult dogs (aged > 1 year and < 10 years) included in the study. For heart rate, a segmented linear regression was applied over two weight intervals (0–20 kg and 21–80 kg).

**Figure 8:**
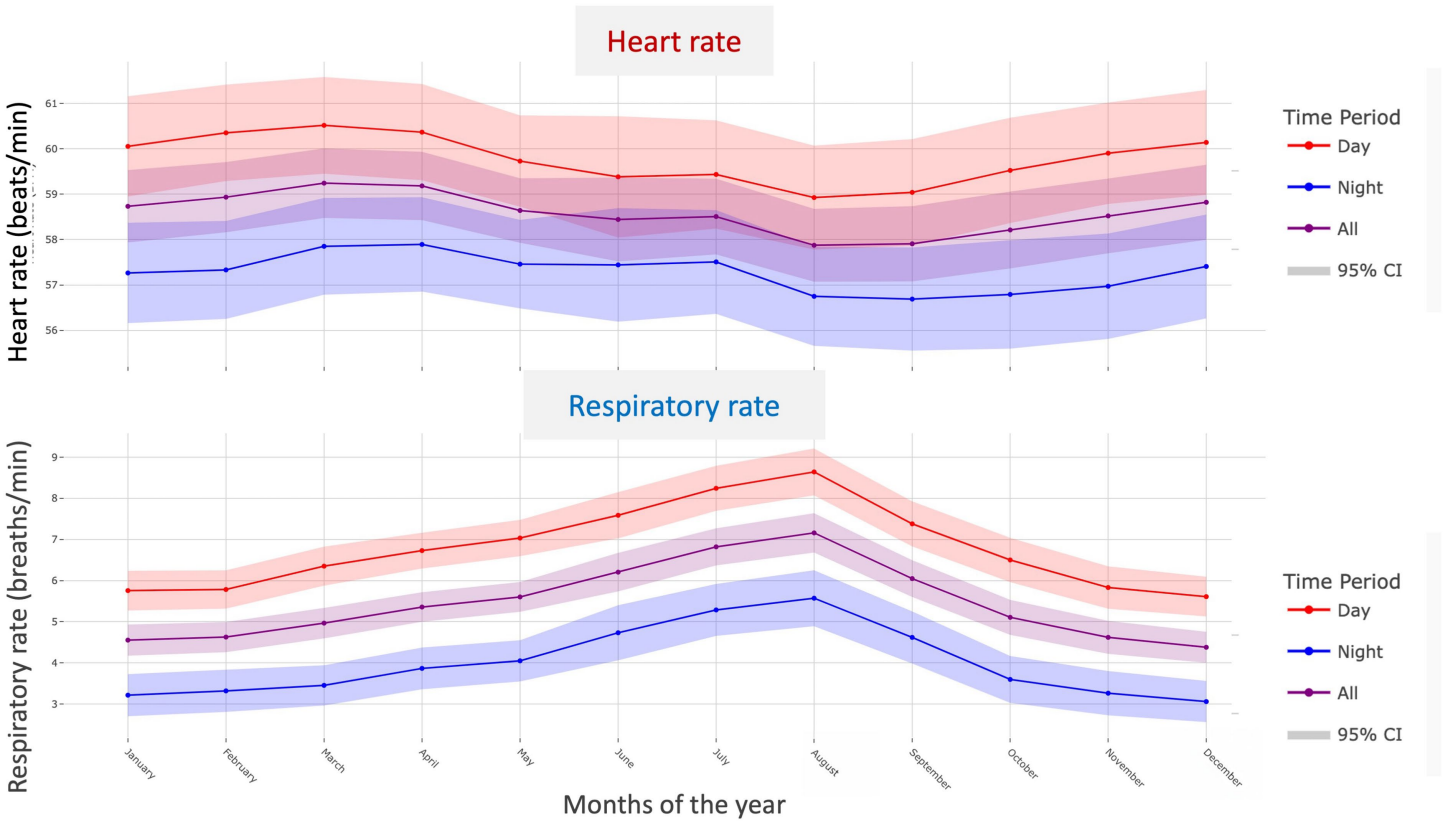
Monthly variations in resting heart rate **(A)** and respiratory rate **(B)** recorded during daytime (red), nighttime (blue), and across the full 24-h period (purple), with 95% confidence intervals, in 121 apparently healthy adult dogs (aged > 1 year and < 10 years) with at least 10 consecutive months of recordings for both variables.

Most dogs (*n* = 533/562, 94.8%) kept the device continuously, both day and night, enabling the collection of day and sleep data over a full 24-h cycle. Results for resting HR and RR according to time of measurement (day *versus* night), sex, body weight, and dog size category in the subpopulation of 562 AH adult dogs (aged > 1 and < 10 years) are presented in Table 1 and Figures 5–8.

**Table 1:** Resting heart rate and respiratory rate according to sex, time of measurement, and body size in 562 apparently healthy adult dogs (aged > 1 and < 10 years), and in comparison with a diseased canine population of 166 adult dogs, including 129 with heart diseases (see text for details).

**Characteristics**	**Sub-groups**	**Number of dogs**	**Heart rate** **(beats/min)**	***P-*value** **(for heart rate)**	**Respiratory rate** **(breaths/min)**	***P-*value** **(for respiratory rate)**
Sex	Male dogs	314	60.3 [54.7–65.2]	0.164	16.0 [13.7–18.4]	0.849
	Female dogs	248	60.7 [56.2–65.4]		16.1 [14.0–18.7]	
Time of measurement *	Nighttime	533	58.3 [53.6–64.2]	**< 0.0001**	14.2 [12.1–17.0]	**< 0.0001**
	Day time	533	61.3 [56.3–66.7]		17.4 [15.2–19.8]	
Dog size category	Large-sized dogs (> 20 kg)	355	59.5 [54.7–63.8]	**< 0.05**	15.6 [13.7–18.4]	**< 0.05**
	Small- to medium-sized dogs (≤20 kg)	207	62.1 [56.4–68.1]		16.5 [14.5–19.0]	
	Medium to large-sized dogs (> 10 kg) Small-sized dogs (≤10 kg)	478	59.5 [54.6–64.2]	**< 0.0001**	15.8 [13.7–18.3]	**< 0.05**
		84	65.0 [60.9–69.4]		17.2 [15.0–19.9]	
Health status	Apparently healthy dogs	562	60.5 [55.2–65.3]	**0.0001**	16.1 [13.8–18.7]	**0.0001**
	Diseased dogs	166	65.4 [60.0–78.5]		17.5 [14.6–20.9]	
	Apparently healthy dogs	562	60.5 [55.2–65.3]	**< 0.0001**	16.1 [13.8–18.7]	**< 0.0001**
	Dogs with heart diseases	129	67.3 [61.0–81.5]		18.0 [14.9–21.5]	

Continuous data are presented as median [interquartile range]. Bold values indicate statistically significant *p-*values. * For the purpose of this study, nighttime was defined as midnight to 6:00 a.m., and daytime as all other hours. Both daytime and nighttime measurements were available for 533 apparently healthy adult dogs out of the 562 included in the study.

A weak but statistically significant positive correlation was found between HR and RR (*r* = 0.285; *p* < 0.0001; Figure 5). No difference in either HR or RR was found between males and females (Table 1). Conversely, both HR and RR were significantly lower at night (*p* < 0.0001; Table 1 and Figure 6) and in large-sized dogs (> 20 kg) as compared to small- and medium-sized dogs (≤ 20 kg; *p* < 0.05; Table 1). Average resting HR and RR values according to body weight are presented in Figures 7A,B, respectively. A weak overall negative correlation was found between RR and body weight (*r* = − 0.111, *p* < 0.05), with a median RR of 16.1 breaths/min [13.8–18.7] (Figure 7B). For HR, a segmented linear regression was applied over two body weight intervals: [0–20] kg and [21–80] kg (Figure 7A). In dogs weighing 20 kg or less, a moderate negative correlation was found between weight and HR (*r* = − 0.330, *n* = 207, *p* < 0.0001), with a median HR of 62.1 bpm [56.4–68.1]. In heavier dogs, a weak but significant positive correlation emerged (*r* = 0.207, *n* = 355, *p* < 0.0001), with a slightly lower median HR value of 59.5 bpm [54.7–63.8]. Additionally, dogs weighing 10 kg or less had significantly higher resting HR and RR than heavier dogs (*p* < 0.0001 and *p* < 0.05, respectively).

To effectively assess the impact of seasonality on resting HR and RR, and to ensure the reliability of the analysis, a specific subset of dogs living exclusively in the Northern Hemisphere was selected from the adult non-senior AH population (*n* = 562). To be included in this seasonal analysis, dogs were required to have at least 10 consecutive months of recordings for both HR and RR, with a minimum of 10 recording days per month and at least 15 min of data per day. A total of 121 AH adult dogs met these criteria and were included in the seasonality analysis. A linear mixed-effects model was used to evaluate monthly variations in both HR and RR. The corresponding results are presented in Figure 8. For HR, several months showed significantly lower values compared to January. In particular, HR was significantly reduced in August (*p* = 0.008), September (*p* = 0.002), and October (*p* = 0.014). No significant differences were observed for the other months (all *p* values > 0.2). In contrast, RR increased significantly from April onwards (*p* < 0.001), peaked in August (*p* < 0.001), and gradually declined through September (*p* < 0.001) and October (*p* = 0.001). Values returned to winter baseline levels in November and December (both *p* > 0.4).

Breed-specific characteristics of resting HR and RR are detailed in Table 2 for the seven most represented breeds (each with at least 15 dogs) among the 562 AH adult dogs included in the study. Golden retrievers (*p* = 0.026), Australian Shepherds (*p* < 0.001), and Border Collies (*p* < 0.01) had significantly lower resting HR values compared to the overall population. For RR, Australian Shepherds showed a significantly higher values (*p* < 0.01), whereas Dobermans had a significantly lower values (*p* < 0.001).

**Table 2:** Breed-specific resting heart rate and respiratory rate characteristics in the seven most represented canine breeds (each with > 15 dogs) among the 562 apparently healthy adult dogs (aged > 1 and < 10 years).

Breed	Number of dogs	Biometric device wear time (days)	Total measured time (hours)	HR (beats/min)	*P* value (for HR)	Delta HR	RR (breaths/min)	*P* value (for RR)	Delta RR	Age (years)	Body weight (kg)
Golden Retriever	31	197.0 [89.5–470.5]	164.1 [36.4–234.0]	57.9 [54.7–60.7]	**0.026**	−1.1	16.0 [13.7–17.6]	0.144	−0.4	2.8 [1.5–4.9]	34.0 [28.0–35.0]
German Shepherd	29	272.0 [30.0–669.0]	189.8 [13.6–323.2]	58.1 [54.1–62.4]	0.131	−0.7	16.3 [14.4–18.7]	0.703	0.5	3.4 [2.0–5.5]	35.0 [30.0–40.0]
Labrador Retriever	26	169.5 [93.5–287.5]	139.1 [39.5–177.2]	57.9 [54.4–63.7]	0.423	−0.1	14.8 [13.2–17.3]	0.110	−0.7	4.5 [3.3–6.7]	31.5 [28.5–37.4]
Australian Shepherd	25	278.0 [69.0–424.0]	146.4 [36.3–200.1]	54.5 [52.5–58.8]	**< 0.001**	−3.2	18.2 [16.9–20.0]	**< 0.01**	2.6	3.8 [2.3–5.7]	23.0 [21.0–25.4]
Border Collie	20	206.5 [77.5–353.8]	107.7 [24.0–159.6]	56.6 [52.4–60.1]	**< 0.01**	−1.7	17.1 [15.1–19.5]	0.082	1.2	2.7 [2.0–4.0]	19.0 [16.0–20.4]
Doberman	18	83.0 [28.2–324.8]	143.3 [14.3–123.4]	60.6 [57.1–64.1]	0.809	1.5	13.8 [11.3–14.7]	**< 0.001**	−2.6	3.1 [1.3–5.5]	36.0 [30.8–38.0]
Siberian Husky	18	277.5 [110.2–513.5]	165.8 [33.6–272.3]	58.1 [55.4–61.9]	0.399	0.0	14.8 [12.5–17.8]	0.059	−1.2	3.2 [2.1–4.2]	22.0 [20.2–27.8]

For each breed, the number of dogs, device wear time (in days), total measured time (in hours), resting heart rate (HR), and respiratory rate (RR) are presented as median [interquartile range], along with age and body weight. The difference between each breed’s median value and the overall population median is reported as Delta HR and Delta RR, respectively. Associated *p* values indicate statistical significance. Bold values denote statistically significant differences.

### 3.4 Comparison of HR and RR between adult non-senior AH dogs (*n* = 562) and adult non-senior diseased dogs (*n* = 166)

Among the 1,012 dogs with complete health questionnaires and available epidemiological data (age, sex, breed, and body weight), 283 were excluded based on the presence of at least one clinical sign reported by their owners, in order to define the AH study population (Figure 1). Among the 283 excluded dogs, 166 were adult non-senior dogs (aged > 1 year and < 10 years) and served as the diseased reference population. Within this subgroup, 129 dogs were diagnosed with various cardiac conditions, including DMVD (*n* = 30), dilated cardiomyopathy (DCM, *n* = 22), and other heart diseases (*n* = 77) with (*n* = 41) or without (n = 88) CHF. Additional reported pathological conditions included motor disorders (*n* = 17) and various others (*n* = 20), such as neurological, dermatological, or cancers.

**Figure 9:**
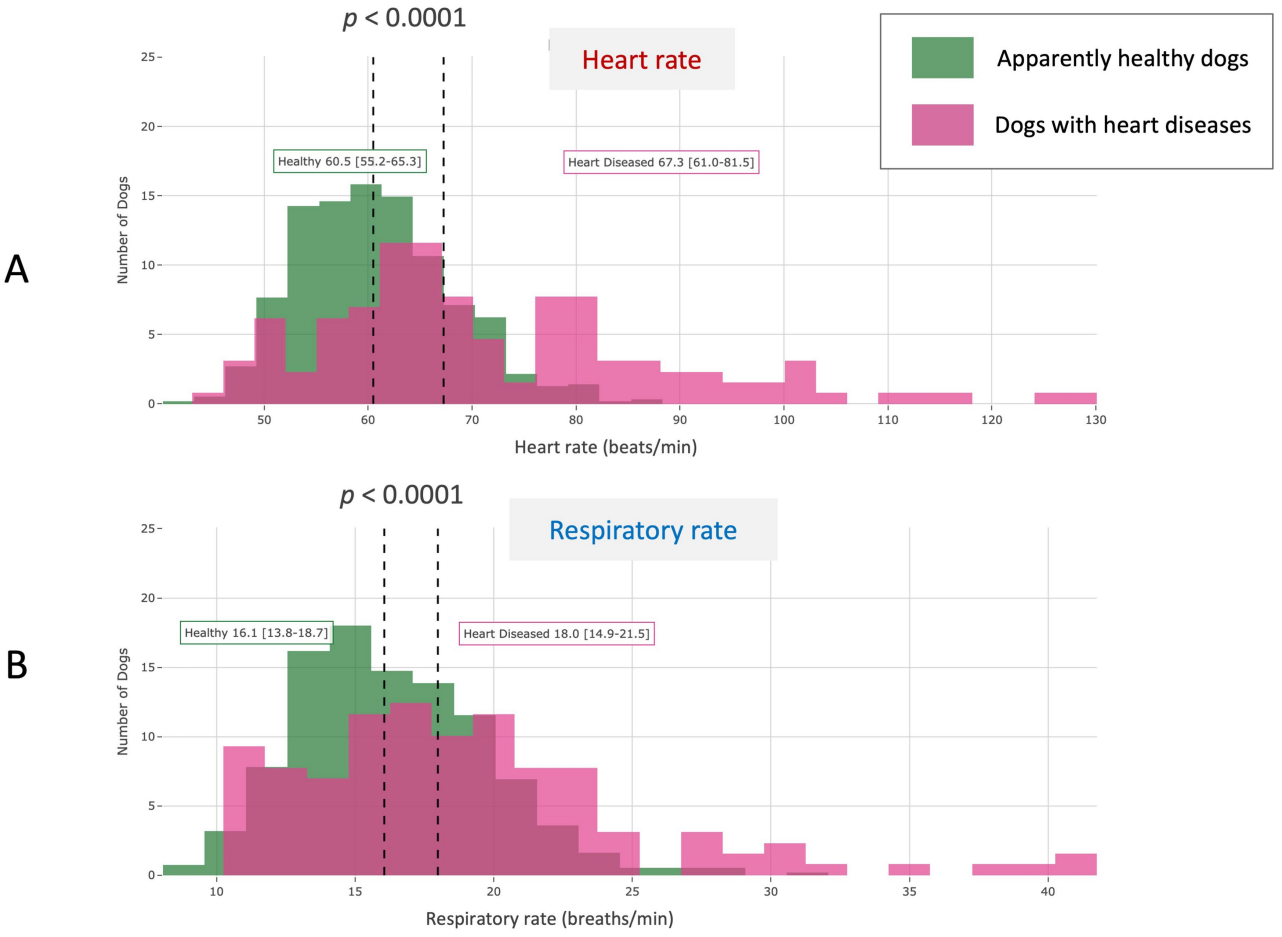
Comparative population distribution of average monthly resting heart rate **(A)** and respiratory rate **(B)** in the 562 apparently healthy adult dogs (aged > 1 year and < 10 years) included in the study and in 129 adult dogs (aged > 1 year and < 10 years) with various heart disorders reported by the owner in the medical questionnaire. Both biometric variables were significantly higher in the diseased group as compared to the healthy group (*p* < 0.0001).

Adult non-senior diseased dogs (*n* = 166) had significantly higher median HR (*p* = 0.0001) and RR (*p* = 0.0001) compared to adult non-senior AH dogs (*n* = 562; Table 1). Similar results were found in the subgroup of adult non-senior dogs with heart diseases (*n* = 129; *p* < 0.0001; Table 1 and Figure 9).

## 4 Discussion

The primary objective of this prospective observational study was to establish a unique database of two vital parameters, HR and RR, recorded over extended periods (i.e., up to several months) at rest, whether awake or asleep, using a biometric health-monitoring device in a large international cohort of AH dogs. To the best of our knowledge, this report represents the first large-scale, long-term, real-world prospective study providing an in-depth analysis of resting HR and RR in several hundred AH dogs (*n* = 703) from a wide variety of breeds. Conducted under everyday home conditions using a validated smart biometric tool, this study generated an unprecedented dataset on canine resting HR and RR, with a detailed examination of the effects of intrinsic (age, sex, weight, breed) and extrinsic (circadian rhythm, season) factors on these vital signs, thus offering novel insights into canine cardiorespiratory physiology under natural conditions.

Cardiac diseases are common in the canine population, affecting approximately 10% of dogs seen in primary care veterinary clinics repetition (17). Among these, DMVD is the leading cause, accounting for about 75% of reported cases, while DCM ranks as the second most common canine acquired heart disease (17, 18). Similarly to various other cardiac disorders, as DMVD and DCM progress, increases in both HR and RR are typically observed (17–19). This underlies the recommendation of the *American College of Veterinary Internal Medicine* (ACVIM) consensus statement on DMVD, which encourages participation in a structured, home-based extended care program that promotes regular monitoring of HR and RR in dogs with CHF (17).

Pulmonary edema, a hallmark of left-sided CHF, is consistently characterized, regardless of its cardiac origin, by restrictive dyspnea, with tachypnea being the most prominent clinical sign. For both DMVD and DCM dogs, home monitoring of RR has been shown to be more reliable in assessing treatment response for left-sided CHF than selected Doppler echocardiographic indices, the cardiac biomarker NT-proBNP, or even RR measurements performed in the clinical setting (19). Sleeping and resting RR measurements are thus commonly used to monitor dogs with left-sided cardiac diseases and to identify those with left-sided CHF (20). Currently, RR is usually monitored at home manually by owners, either with a stopwatch or, increasingly, with the aid of dedicated smartphone applications that are free and user-friendly. However, these manual measurements have several limitations: they are point-in-time readings, typically based on 30-s intervals, thus representing less than 0.1% of a 24-h period. As such, they fail to capture RR daily variations and the effects of medication on RR values throughout the day. This highlights the relevance of using a portable smart device, such as the one used in the present study, which enables automated monitoring of RR both during the day and also at night, offering a more accurate and comprehensive picture of a dog’s respiratory status.

Similarly, HR is a relevant follow-up parameter in dogs with heart diseases, as it typically increases during episodes of cardiac decompensation. For example, in DMVD, multiple studies have shown that HR is significantly higher in dogs with CHF (ACVIM stage C or D) compared to those at ACVIM stage B (19, 21, 22). Conversely, the resolution of cardiogenic pulmonary edema is usually associated with a decrease or even normalization of HR. This underscores the relevance of monitoring HR, particularly by owners at home, as this setting helps eliminate the stress effect commonly observed in clinical environments. However, as with RR, home HR monitoring by dog owners has several limitations. These are point-in-time sample-based measurements that are not representative of HR fluctuations throughout the day. In addition, HR measurement is more technically challenging than RR measurement for dog owners, as it requires either precordial palpation or the use of a stethoscope, which most are not trained to use properly. This once again highlights the relevance of automated HR monitoring made possible by smart biometric devices, such as the one used in the present study, which also enable nighttime recordings.

Our study presents several key strengths. First, it was a prospective study with strict inclusion criteria designed to ensure data reliability. In addition to requiring that owners complete a detailed medical questionnaire confirming the dog’s apparent good health, only dogs with complete metadata (age, sex, breed, and body weight) were retained. Furthermore, dogs showing clinical signs or statistical abnormalities during the study period were systematically excluded. Another important strength lies in the fact that all dogs were monitored either awake or asleep in their natural environment rather than in a clinical setting, thanks to the use of a non-invasive smart biometric device easily attached to the dog’s regular collar, allowing easy incorporation into daily life without disrupting the dog’s routine. As a result, the median device wear time was notably long (approximately 6 months) enabling the collection of robust longitudinal data. In addition, the large sample size (703 AH dogs), combined with high epidemiological diversity, characterized by 113 different pure breeds, a wide range of body weights (0.9–75.0 kg), and ages (0.2–18.1 years), provided a solid basis for statistical subgroup analysis.

Altogether, the combination of strict inclusion criteria, long-term real-life monitoring, large and diverse cohort, and prolonged recording periods led to the generation of a high-quality physiological dataset, highlighting how HR and RR may vary according various key physiological and environmental factors, such as age, body weight, breed, time of day, and season.

Our results demonstrate that age is a major determinant of both HR and RR in dogs, especially during early life stages (i.e., before 1 year of age). During this period, we observed a strong negative correlation between age and both HR and RR. The early decline in HR is consistent with previous studies using auscultation, standard electrocardiogram recordings, blood pressure or 24-h Holter monitoring, which have reported higher HR values in healthy dogs under 1 year of age compared to healthy adults (23–27). This finding may be explained by the physiological characteristics of cardiac autonomic regulation in neonates and puppies, notably an increased sensitivity to exogenous norepinephrine and a lower resting vagal tone compared to adult dogs, and also by increased body size over time (28).

Conversely, adult dogs in the present study showed a very weak positive correlation between HR and age from one to 10 years, suggesting relative stability during this life stage with only a slight age-related increase. Interestingly, this increase became more pronounced in senior dogs, as a weak but statistically significant rise in HR was observed in animals older than 10 years. Several studies conducted in clinical settings have similarly reported that HR tends to be lowest in young adult dogs and increases in older dogs (23, 24, 26, 27). This may be explained by a direct effect of aging on HR, possibly related to age-associated changes in autonomic balance and decreased baroreflex sensitivity in geriatric dogs (29), as demonstrated in humans (30, 31). However, the potential contribution of subclinical cardiac or systemic conditions, characterized by reduced parasympathetic activity and increased sympathetic tone and more likely to emerge with advancing age, cannot be ruled out.

In our cohort, a strong negative correlation between age and RR was also observed during early life, followed by a very weak positive correlation after 12 months of age, indicating only a slight increase over time. In a study involving AH dogs aged 7 to 183 months (median: 59 months), sleeping RR was reported to be unaffected by age (32). Similarly, RR measured in a clinical setting did not differ significantly between puppies and adult dogs in another report (27). However, RR values in our study were obtained under different conditions: measurements were collected non-invasively in the dogs’ natural environment, rather than in a veterinary clinic, and not exclusively during sleep. Instead, data were recorded when the dog was simply immobile, potentially reflecting a broader range of resting physiological states. The elevated resting RR observed in puppies in our study may be attributed to immature respiratory control mechanisms, smaller lung volumes, and higher metabolic rates during early development, as has been well documented in human infants and children (33).

In mammals, HR scales allometrically with body size, decreasing as body mass increases. This inverse relationship leads to markedly lower HR values in large species such as whales (less than 10 bpm during dives and approximately 20 to 30 bpm at the surface), and substantially higher HR values in small mammals like rodents, which may exceed 500 to 700 bpm (34, 35). In clinical settings, HR has also been shown to be negatively associated with body weight in dogs, with a mean HR difference of 10.5 bpm between a 5 and a 55 kg dog (23), although some studies do not support a correlation between HR and body weight in AH dogs (24, 36). In the present study, resting HR was significantly lower in large-sized adult AH dogs (> 20 kg) than in small- and medium-sized adult AH dogs (≤ 20 kg), with a moderate negative correlation between body weight and HR in the latter, but, surprisingly not in the former. A weak overall negative correlation was also found between resting RR and body weight. Despite reaching statistical significance, this finding suggests that body weight has only a limited impact on resting RR in AH dogs during adulthood.

In our study, the effect of the time of measurement was also investigated. As most dogs (94.8%) wore the device continuously, both during the day and at night, this allowed for the collection of data across the entire 24-h cycle, including both wake and sleep periods. This ensured a balanced temporal distribution of the recorded physiological signals and minimized circadian sampling bias. Both resting HR and RR were found to be significantly lower during nighttime compared to daytime. Importantly, in our real-life conditions, dogs were not required to be asleep for data to be collected. Despite this, clear circadian variations were observed for both parameters, supporting the robustness of intrinsic daily rhythms in dogs under natural conditions. Our findings regarding nocturnal HR are consistent with previous electrocardiogram and Holter-based studies that have documented the influence of the sleep–wake cycle on autonomic regulation in dogs, particularly in relation to HR variability (37–39). As in humans, this nighttime decrease is attributed to enhanced parasympathetic tone and reduced sympathetic activity during sleep (37).

To the best of our knowledge, no study has specifically characterized circadian variations in resting RR in healthy dogs. Previous investigations have focused primarily on RR measurements during sleep or rest, without comparing daytime and nighttime values (20, 32, 40). The present study provides novel insights into daily RR fluctuations under real-life conditions, thereby expanding current knowledge of canine respiratory physiology. Similar to HR, the nocturnal decrease in RR observed in our study may reflect reduced metabolic demand and increased vagal modulation of respiratory drive during sleep. Future studies are needed to determine whether daytime or nighttime values of HR and RR, or their relative variations, offer the highest sensitivity and specificity for the early detection of subclinical heart diseases and adverse cardiorespiratory events in dogs.

To investigate seasonal effects on resting HR and RR in the present study, only adult AH dogs living in the same hemisphere (Northern Hemisphere) and monitored over a long period (≥ 10 consecutive months) were included in the analysis (*n* = 121) to ensure data reliability. Our findings highlight distinct seasonal patterns for resting HR and RR, with a notable decrease in HR and, conversely, a marked increase in RR during the summer months. The rise in RR is consistent with the development of thermal polypnea, which plays a key role in canine thermoregulation through evaporative heat loss (41). The decrease in HR, also reported in humans in the supine position (42), may result from a combination of physiological mechanisms, including reduced basal metabolic demands during warmer periods, possibly due to increased sleep or lower activity levels, along with enhanced parasympathetic tone during deeper or more prolonged rest phases. In addition, higher ambient temperatures can promote peripheral vasodilation, which reduces cardiac preload and afterload, thereby lowering overall cardiac workload under resting conditions. It should be noted that vasodilation can also elicit reflex tachycardia, particularly in response to postural changes, as described in thermophysiological studies in humans (42). However, in the present study, measurements were automatically recorded by the device only when the dog was lying down and immobile.

In the present study, breed-related differences in resting HR and RR were also observed. Golden Retrievers, Border Collies and Australian Shepherds had significantly lower resting HR compared to the overall population, while Australian Shepherds showed a significantly higher RR. Lower HR in Golden Retrievers and Border Collies is consistent with previous reports (23), in which several working breeds (including Border Collies, Golden and Labrador Retrievers) exhibited lower HR, possibly due to selective breeding for superior exercise capacity and cardiovascular fitness, or because these breeds are often chosen by owners with active lifestyles, leading to higher physical activity levels and improved cardiovascular conditioning in the dogs. The elevated RR in Australian Shepherds, despite moderate body size, may relate to their high activity levels and herding drive, potentially reflecting a more responsive respiratory control system.

Dobermans exhibited the lowest RR, which could be associated with their large body size and deep chest conformation, traits previously linked to lower HR and efficient cardiovascular mechanics in large deep-chested breeds, and potentially contributing to more efficient ventilatory function (26). While the present study was not designed to investigate causal mechanisms, these findings highlight the potential value of breed-specific reference intervals when interpreting resting biometric parameters.

In a complementary analysis, we investigated whether continuous biometric monitoring could distinguish AH dogs from those affected by various diseases. A total of 166 adult non-senior dogs served as the diseased reference population, including 129 diagnosed with cardiac conditions such as DMVD and DCM. Compared to age-matched AH dogs (*n* = 562), diseased dogs (*n* = 166) exhibited significantly higher median resting HR and RR. These differences remained significant when the analysis was restricted to dogs with heart diseases. The observed shifts in HR and RR distributions suggest that long-term, at-home biometric monitoring can detect abnormal patterns associated with cardiac disorders. This opens promising avenues for the development of AI-based tools for early detection and longitudinal monitoring of canine cardiorespiratory diseases, as illustrated in Figure 2. Among the 26 excluded dogs, one developed pulmonary edema due to decompensated DMVD. Interestingly, this CHF event was preceded by a progressive increase in resting HR over a 120-day period, which remained clinically silent and went unnoticed by the owner. This early HR signal was detected by the algorithm several weeks before the onset of overt clinical signs (notably dyspnea with increase in resting RR), suggesting that such biometric trends could represent prodromal markers of impending decompensation. This case highlights that daily remote cardiorespiratory monitoring with AI support can detect health deterioration before visible signs appear, potentially allowing earlier action and improved care.

This study presents several limitations. First, the AH status of the dogs was based solely on owner-reported information collected through a dedicated medical questionnaire, without clinical examination or cardiac auscultation by a veterinarian. Consequently, the inclusion of dogs with subclinical DMVD (ACVIM stage B1 or even B2) or early-stage DCM (stage B1 or B2) cannot be ruled out (17, 18). Moreover, other subclinical, non-cardiac conditions (e.g., endocrine, metabolic, respiratory, or inflammatory diseases) could also have been present without the owners’ awareness and may have influenced resting HR and RR. Second, “nighttime” was defined in this study as midnight to 6:00 a.m. to allow standardized comparisons across the international cohort. However, this may not correspond to the actual sleep period of all dogs. Future studies incorporating accelerometer-based or owner-reported sleep–wake data could refine the analysis of canine HR and RR circadian variations. Furthermore, although the study included dogs with a wide body weight range (0.9 to 75.0 kg), most were medium- to large-sized, with a median body weight of 23.0 kg. This may have resulted in an underrepresentation of dogs weighing less than 10 kg, potentially limiting the precision and applicability of the findings to this subgroup, which represents a substantial proportion of the canine population in urban settings. Lastly, although the biotracker does record HR variability, which is an important marker of autonomic nervous system influence on cardiac function typically evaluated via 24-h Holter monitoring, the present analysis deliberately focused only on combined instantaneous resting HR and RR values (43, 44). Nevertheless, the biometric device used here does not assess cardiac electrical activity and is therefore not intended to replace Holter monitoring, which remains the gold standard for arrhythmia detection in dogs, both at rest and during activity (43–46). However, Holter recordings do not provide RR measurements, a key strength of the biometric device used in this study. Thus, despite the above-mentioned limitations, this wearable health tracker offers a non-invasive, long-term, real-life monitoring solution that is uniquely suited to large-scale studies and to day-and-night tracking of combined HR and RR under everyday conditions, an approach not currently achievable with traditional monitoring systems.

## 5 Conclusion

To the best of our knowledge, this report represents the first large-scale, real-life, prospective study providing an in-depth, long-term concomitant analysis of resting HR and RR in 703 AH awake or asleep dogs using a biometric health tracker. It revealed that key factors, including age, body weight, circadian rhythm, season, and breed, can significantly influence these two vital parameters. This unique reference database substantially advances our understanding of normal canine cardiorespiratory physiology. It also lays the groundwork for the early detection of pathological deviations through statistical or AI data-driven approaches, opening new perspectives for improved monitoring of at-risk dogs and optimized, even anticipatory, therapeutic strategies in those with confirmed pathological conditions.

Part of this study was presented as an oral communication at the 5th annual Numanima meeting on June 25, 2025 (Maisons-Alfort, France).

## Data Availability

The original contributions presented in the study are included in the article/supplementary material, further inquiries can be directed to the corresponding author.
