# Correction: Resting heArt and respIratory rates in dogs in their natural environment: new insights from a long-term, international, prospective study in a COhort of 703 dogs using a biometric device for LongitudinaL non-invasive cARdiorespiratory monitoring (the AI-COLLAR study)

**DOI:** 10.3389/fvets.2025.1711869

**Published:** 2025-10-30

**Authors:** Valérie Chetboul, Eric Humbert, Louis Dougoud, Guillaume Lorre

**Affiliations:** ^1^École Nationale Vétérinaire d'Alfort, Maisons-Alfort, France; ^2^INSERM, IMRB, Univ Paris Est Créteil, Créteil, France; ^3^Invoxia, Issy-les-Moulineaux, France

**Keywords:** artificial intelligence, cardiology, canine, heart disease, cardiac physiology

The title of this article was erroneously given as: **Resting heart and respiratory rates in dogs in their natural environment: new insights from a long-term, international, prospective study in a cohort of 703 dogs using a biometric device for longitudinal non-invasive cardiorespiratory monitoring (the AI-COLLAR study)**.

The correct title of the article is **Resting heArt and respIratory rates in dogs in their natural environment: new insights from a long-term, international, prospective study in a COhort of 703 dogs using a biometric device for LongitudinaL non-invasive cARdiorespiratory monitoring (the AI-COLLAR study)**.

The acronym “AI-COLLAR” in the incorrect article title was not highlighted as originally intended. The title now includes the capital letters needed to build the acronym AI-COLLAR.

The original version of this article has been updated.

